# Human liver derived mesenchymal stromal cells ameliorate murine ischemia-induced inflammation through macrophage polarization

**DOI:** 10.3389/fimmu.2024.1448092

**Published:** 2024-07-22

**Authors:** Yun Liang, Elif Ozdogan, Michael J. Hansen, Hui Tang, Ishran Saadiq, Kyra L. Jordan, James D. Krier, Deep B. Gandhi, Joseph P. Grande, Lilach O. Lerman, Timucin Taner

**Affiliations:** ^1^ Department of Surgery, Mayo Clinic, Rochester, MN, United States; ^2^ Boston Children’s Hospital, Harvard Medical School, Boston, MA, United States; ^3^ Department of Immunology, Mayo Clinic, Rochester, MN, United States; ^4^ Division of Nephrology and Hypertension, Mayo Clinic, Rochester, MN, United States; ^5^ Department of Laboratory Medicine and Pathology, Mayo Clinic, Rochester, MN, United States

**Keywords:** mesenchymal stromal cells, immunomodulation, renal artery stenosis, liver tolerance, inflammation

## Abstract

**Introduction:**

The immunomodulatory properties of mesenchymal stromal cells (MSC) have been well-characterized in *in-vitro* and *in-vivo* models. We have previously shown that liver MSC (L-MSC) are superior inhibitors of T-cell activation/proliferation, NK cell cytolytic function, and macrophage activation compared to adipose (A-MSC) and bone marrow MSC (BM-MSC) *in-vitro*.

**Method:**

To test these observations *in-vivo*, we infused these types of MSC into mice with unilateral renal artery stenosis (RAS), an established model of kidney inflammation. Unilateral RAS was induced via laparotomy in 11-week-old, male 129-S1 mice under general anesthesia. Control mice had sham operations. Human L-MSC, AMSC, and BM-MSC (5x105 cells each) or PBS vehicle were injected intra-arterially 2 weeks after surgery. Kidney morphology was studied 2 weeks after infusion using micro-MRI imaging. Renal inflammation, apoptosis, fibrosis, and MSC retention were studied *ex-vivo* utilizing western blot, immunofluorescence, and immunohistological analyses.

**Results:**

The stenotic kidney volume was smaller in all RAS mice, confirming significant injury, and was improved by infusion of all MSC types. All MSC-infused groups had lower levels of plasma renin and proteinuria compared to untreated RAS. Serum creatinine improved in micetreated with BM- and L-MSC. All types of MSC located to and were retained within the stenotic kidneys, but L-MSC retention was significantly higher than A- and BM-MSC. While all groups of MSC-treated mice displayed reduced overall inflammation and macrophage counts, L-MSC showed superior potency *in-vivo* at localizing to the site of inflammation and inducing M2 (reparative) macrophage polarization to reduce inflammatory changes.

**Discussion:**

These *in-vivo* findings extend our *in-vitro* studies and suggest that L-MSC possess unique anti-inflammatory properties that may play a role in liver-induced tolerance and lend further support to their use as therapeutic agents for diseases with underlying inflammatory pathophysiology.

## Introduction

Mesenchymal stromal cells (MSC) have been widely studied for their potential as therapeutic agents to treat a multitude of inflammatory pathologies due to their immunomodulatory capabilities. MSC have been derived from several types of tissues, but those isolated from adipose tissue and bone marrow are most often used in clinical trials. Guided by the liver’s unique tolerogenic microenvironment and immunomodulatory properties, we postulate that liver-derived MSC (L-MSC) may have superior therapeutic potential. In fact, *in-vitro* studies that directly compared MSC isolated from healthy adult liver (L-MSC) to either those from adipose (A-MSC) or bone marrow (BM-MSC) demonstrate that L-MSC are superior at inhibiting the proliferation of alloreactive T cells, IFNy production by T cells (1), and the cytotoxic abilities of NK cells (2). Additionally, transcriptomic and proteomic analyses of A-, BM-, and L-MSC show significantly higher level of expression of several key immunomodulatory molecules in L-MSC (1).

Collectively, the *in-vitro* studies suggest that L-MSC possess a distinct genomic profile that may enhance their immunomodulatory capabilities compared to A- or BM-MSC. The goal of this study is to characterize the function of L-MSC *in-vivo* and evaluate if their superior immunomodulatory capabilities seen *in-vitro* translate into better function *in-vivo*. We examined the therapeutic and immunomodulatory function of L-MSC in the context of ischemic injury using the validated unilateral renal artery stenosis (RAS) mouse model and directly compared their effect to that of A- and BM-MSC. We hypothesized that L-MSC would be non-inferior in their ability to improve overall renal function in the stenotic kidney with greater influence on immunological changes compared to A- or BM-MSC.

## Materials and methods

### Cell culture

The collection of MSC from healthy adults are approved by Mayo Clinic Institutional Review Board (IRB #17-007379 (liver), IRB #11-009182 (adipose tissue) and IRB # 10-002572 (bone marrow). All tissues are collected as part of scheduled donation procedures and informed consent are obtained prior to collecting tissue samples for this study. MSC are isolated and passaged from human adipose, bone marrow, and liver tissue as previously described (1–3). Specifically, adipose tissue is obtained from the subcutaneous compartment during the abdominal incision for a living donor nephrectomy procedure. Bone marrow aspiration from the iliac crest is performed by specialized hematology team under general anesthesia as part of living donor nephrectomy procedure. A liver biopsy sample, measuring 1cm x 1cm, is obtained from donor organs (deceased or living donor) for isolation of MSC. After obtaining tissue samples, the source tissue is enzymatically digested, and the plastic-adherent cells from the resulting cell suspension are placed into MSC culture media and are allowed to proliferate for 2 weeks before first passage. The cell lines used to date represent both sexes (50% female), racial heterogeneity (>10% non-Caucasians), and a wide range of ages from 20 to 75. Their phenotype and trilineage differentiation capacity were confirmed with flow cytometry and MSC functional identification assay (R&D Systems, Minneapolis, MN, USA), respectively. Prior to administration into mice, MSC (5x10^5^ cells in 200ul PBS) in Passage 3 were fluorescently labeled with CellTrace™ Far Red (CTFR, ThermoFisher Scientific, Waltham, MA, USA) to allow for detection after infusion.

### Renal artery stenosis model

All protocols were approved by Mayo Clinic IRB and Institutional Animal Care and Use. As previously described (4), 11-week-old, male 129-S1 mice (Jackson laboratory, Bar Harbor, ME, USA) underwent open laparotomy under general anesthesia. After exposure of the right renal artery, a 0.15mm diameter arterial cuff was placed on the artery and secured with sutures to achieve partial occlusion of blood flow to the right kidney (i.e., stenotic kidney, STK). Two weeks following RAS surgery, fluorescently tagged MSC (5x10^5^ cells in 200ul of PBS) derived from human adipose (A-MSC), bone marrow (BM-MSC), or liver (L-MSC) tissues, were given to RAS mice intra-arterially through direct cannulation of the carotid artery via vascular cut down. Mice that underwent surgery without cuff placement (n=4) served as negative controls (i.e. sham group). Mice that underwent RAS surgery but received an infusion of PBS (n=4) served as positive controls (i.e. untreated RAS group). Tail cuff blood pressures (Kent Scientific, Torrington, CT, USA) were also obtained at baseline, two weeks following RAS surgery, and two weeks following MSC infusion. General anesthesia was achieved using 3% isoflurane inhalation for induction and 1.5% during RAS surgery and intra-arterial MSC injection. Mice were euthanized after MRI imaging. Briefly, mice underwent general anesthesia with isoflurane as stated above. A midline abdominal incision (approximately 1-2cm in length) was made to access the peritoneal cavity. Peritoneal organs were then reflected superiorly to expose the inferior vena cava in order to obtain blood samples. After exsanguination, the STKs were collected for tissue processing.

### Imaging protocol

Two weeks after MSC or PBS injection, mice were scanned using MRI as previously described (5). Previously established imaging protocols were used to acquire the appropriate images to quantify the volume, perfusion, and oxygenation of the STKs (5, 6). All image analyses were performed using Analyze software (version 12.0; Biomedical Imaging Resource, Mayo Clinic, MN, USA) and Matlab (The MathWorks, Natick, MA, USA).

### Serum and urinary biomarker measurements

Post MRI imaging, blood from the inferior vena cava and urine were collected at the time of euthanasia. Whole blood was centrifuged, and the resulting plasma was collected. Plasma renin concentration was measured by the Renin Assay Kit (Cat#MAK157, Millipore Sigma, St. Louis, MO, USA). Serum creatinine was measured using the Serum Creatinine Detection Kits (Cat# KB02-H, Arbor Assays, Ann Arbor, MI, USA). Urinary protein levels were measured using the Pierce™ Bradford Protein Assay kit (Cat#23200, ThermoFisher, Waltham, MA, USA). All kits were used per manufacturer’s instructions.

### Immunohistochemistry

Following imaging, mice were euthanized as described above, and the STKs were collected and divided into equal parts for both frozen and paraffin-embedded sectioning. Paraffin-embedded STK sections were stained with CD45 (overall inflammation, 1:200 dilution, Cat#ab10558, Abcam, Waltham, MA, USA); CD14 (overall macrophage, 1:200 dilution, Cat#ab182032, Abcam); F4/80 (1:100 dilution, Cat#ab6640, Abcam) and iNOS (M1, inflammatory macrophage: 1:100 dilution, Cat#sc-7271, Santa Cruz Biotechnology, Dallas, TX, USA); F4/80 and mannose receptor-1 (M2, reparative macrophage, 1:100 dilution, Cat#HPA004114, Sigma Aldrich, St. Louis, MO, USA); trichrome (fibrosis, Cat#NC9485545, ThermoFisher); TUNEL (apoptosis, Cat#G3250, Promega, Madison, WI, USA); and PAS (renal cortical tubular atrophy, Cat#395B-1KT, Sigma Aldrich). Frozen STK sections were stained with DHE (reactive oxygen species, Cat#D11347, ThermoFisher). All non-diluted antibodies were used per manufacturer instructions. Six images of each stain were captured with Zeiss^®^ microscope for immunofluorescence stains and Nikon^®^ microscope for immunohistochemistry stains. M1 (double positive for F4/80 and iNOS^+^), M2 (double positive for F4/80 and mannose receptor-1^+^), TUNEL^+^, and MSC retention were quantified by manual counts per high power field. Cortical tubular atrophy was scored by adapting the Banff criteria by an independent pathologist who was blinded to the treatment groups using PAS-stained slides (7). All other stains were quantified based on the percentage of positive stain area using ImageJ (8).

### RT-PCR

Frozen STK samples were homogenized in 350ul of ice-cold lysis buffer, supplied by mirVana PARIS total RNA isolation kit (Cat# AM1556, ThermoFisher Scientific). Total RNAs were then isolated from homogenized samples according to the kit protocol. Total RNA concentrations were measured by a NanoDrop Spectrophotometer (NanoDrop). First-strand cDNA was produced from 800ng of total RNA using SuperScript VILO cDNA Synthesis kit (Cat#11755-050, ThermoFisher Scientific). Relative quantitative PCR were performed using Taqman assays, containing 4ul of cDNA products. All primers were purchased from ThermoFisher Scientific with the following catalog numbers: CD45 (Mm01293577); IFNy (Mm01168134); TNFa (Mm00443258); and GAPDH (Mm99999915). PCR analysis was done on Applied Biosystems Quantstudio 7 using the following conditions: 50°C for 2 minutes, 95°C for 10 minutes and 40 cycles of 95°C for 15 seconds and 60°C for 1 minute. Fold changes of gene expressions were calculated using 2-ΔΔCT method.

### Western blot

Frozen STK samples were homogenized, and protein expression was expressed by western blotting. Protein concentrations were measured using a BCA Protein Assay Kit (Cat# 23225, ThermoFisher Scientific) per manufacturer’s instructions. The membranes were blocked with 5% BSA, incubated with primary antibodies, washed, and incubated with secondary antibodies at room temperature. Finally, the membranes were washed and incubated with ECL Western Blot Substrate (Cell Signaling Technology, Inc., Danvers, MA, USA) and were visualized on ImageQuant™ LAS4000. Anti-IFNy (Cat# BS-0480R, Bioss, Woburn, MA, USA) and anti-TNFa (Cat# ab6671, Abcam, Waltham, MA, USA) antibodies were used as primary antibodies. GAPDH antibody was used to normalize the results.

### Statistical analysis

All statistical analyses were performed using GraphPad Prism version 10.2.2 (324) for Windows (GraphPad Software, Boston, Massachusetts USA, www.graphpad.com). All data are expressed as either mean ± SD for normally distributed data or median [IQR] for non-normally distributed data. Hypothesis testing was carried out using one-way ANOVA followed by a student t-test for normally distributed data. Data not following normal distribution were analyzed using Kruskal-Wallis followed by Wilcoxon test. All data were considered significant if p<0.05.

## Results

### Blood pressure

**Figure 1:**
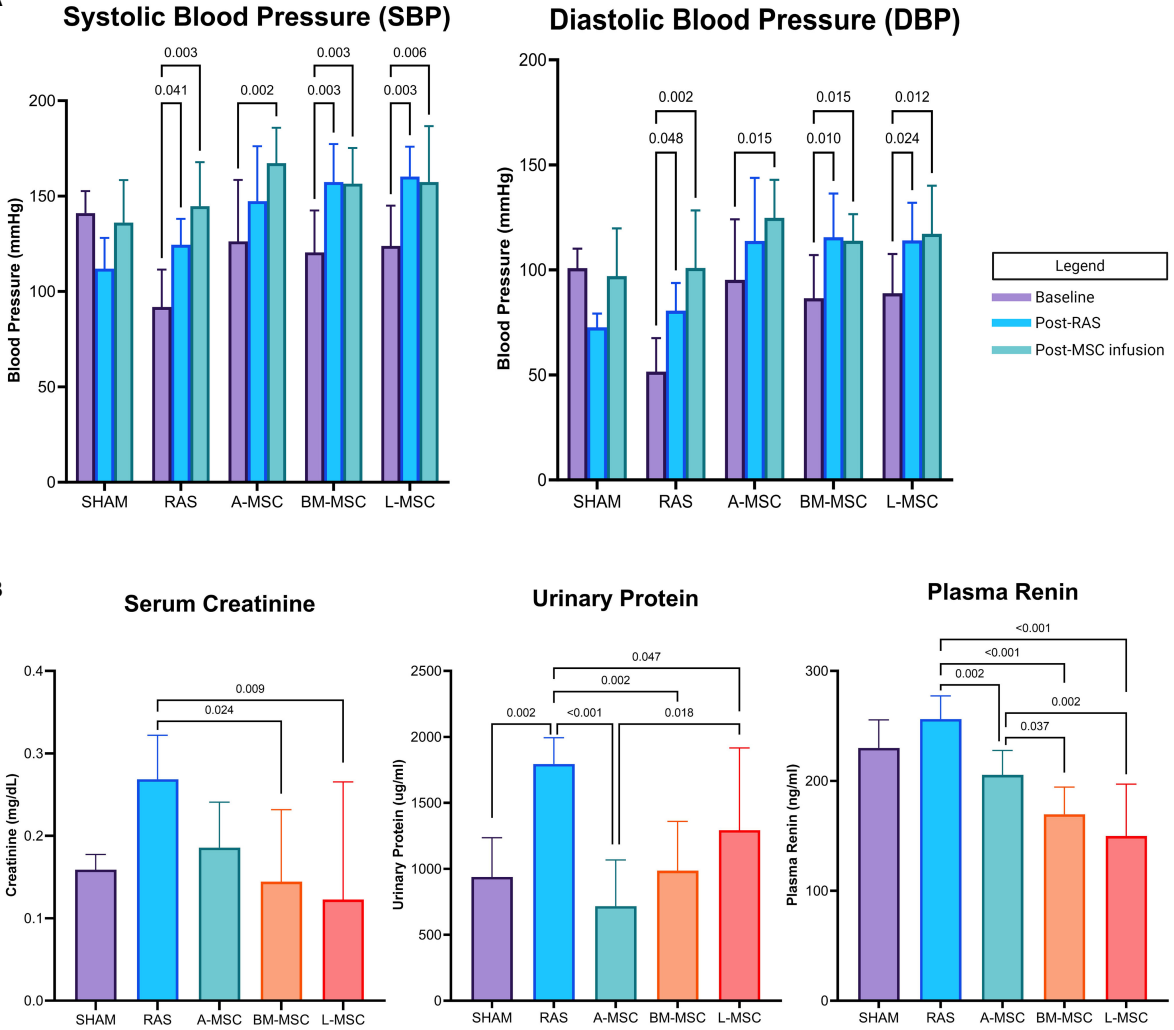
Blood pressure (mean ± SD) measured by tail cuff within each group at baseline, after RAS surgery, and after PBS or MSC infusion are shown in **(A)**. Serum creatinine, urinary protein, and plasma renin levels for each group are shown in **(B)**. All levels are expressed as mean ± SD. RAS, renal artery stenosis.

Initially, eight mice were randomly assigned to receive infusion of each type of MSC. At the conclusion of the study, two mice in the A-MSC group were lost due to total infarction of the STK, one mouse in the BM-MSC was lost due to hydronephrosis of the STK secondary to ureteral stricture, and one mouse in the L-MSC died just prior to MRI imaging, resulting in a final count of A-MSC (n=6), BM-MSC (n=7), and L-MSC (n=7) for analyses. Blood pressure using tail cuffs were obtained at baseline, post-RAS surgery, and post-MSC or PBS infusion. As expected, mean systolic (SBP) and diastolic blood pressure (DBP) measurements were higher than baseline after RAS surgery (**Figure 1A**). Injection of MSC did not show reduction of overall SBP or DBP nor in the amount of absolute or percent change in SBP or DBP from RAS surgery to post-MSC injection (data not shown).

### Serum and urine biomarkers

RAS induced proteinuria (1795 ± 199ug/ml vs 938 ± 297ug/ml in sham, p= 0.002) and tended to elevate serum creatinine (0.27 ± 0.05mg/dL vs 0.16 ± 0.02mg/dL in sham, p= 0.062) compared to sham (**Figure 1B**). Compared to RAS, proteinuria (A-MSC: 717 ± 350ug/ml, p<0.001; BM-MSC: 986 ± 374ug/ml, p= 0.002; L-MSC: 1292 ± 624ug/ml, p= 0.047) and plasma renin (A-MSC: 205.5 ± 22.2ng/ml, p= 0.002; BM-MSC: 169.6 ± 24.7ng/ml, p< 0.001; L-MSC: 149.8 ± 47.2ng/ml, p< 0.001; RAS: 256.3 ± 21.0ng/ml) decreased with MSC treatment for all types. Mice treated with either BM-MSC or L-MSC also resulted in decreased mean serum creatinine (BM-MSC: 0.14 ± 0.09mg/dL, p= 0.024; L-MSC: 0.12 ± 0.14mg/dL, p= 0.009; all vs RAS). Compared to A-MSC, L-MSC treated mice had lower plasma renin levels (149.8 ± 47.2ng/ml vs A-MSC, p= 0.002) but higher proteinuria (1292 ± 624ug/ml vs A-MSC, p= 0.018). No differences were noted among the three MSC groups for serum creatinine (**Figure 1B**).

### Renal volume, perfusion, and oxygenation

**Figure 2:**
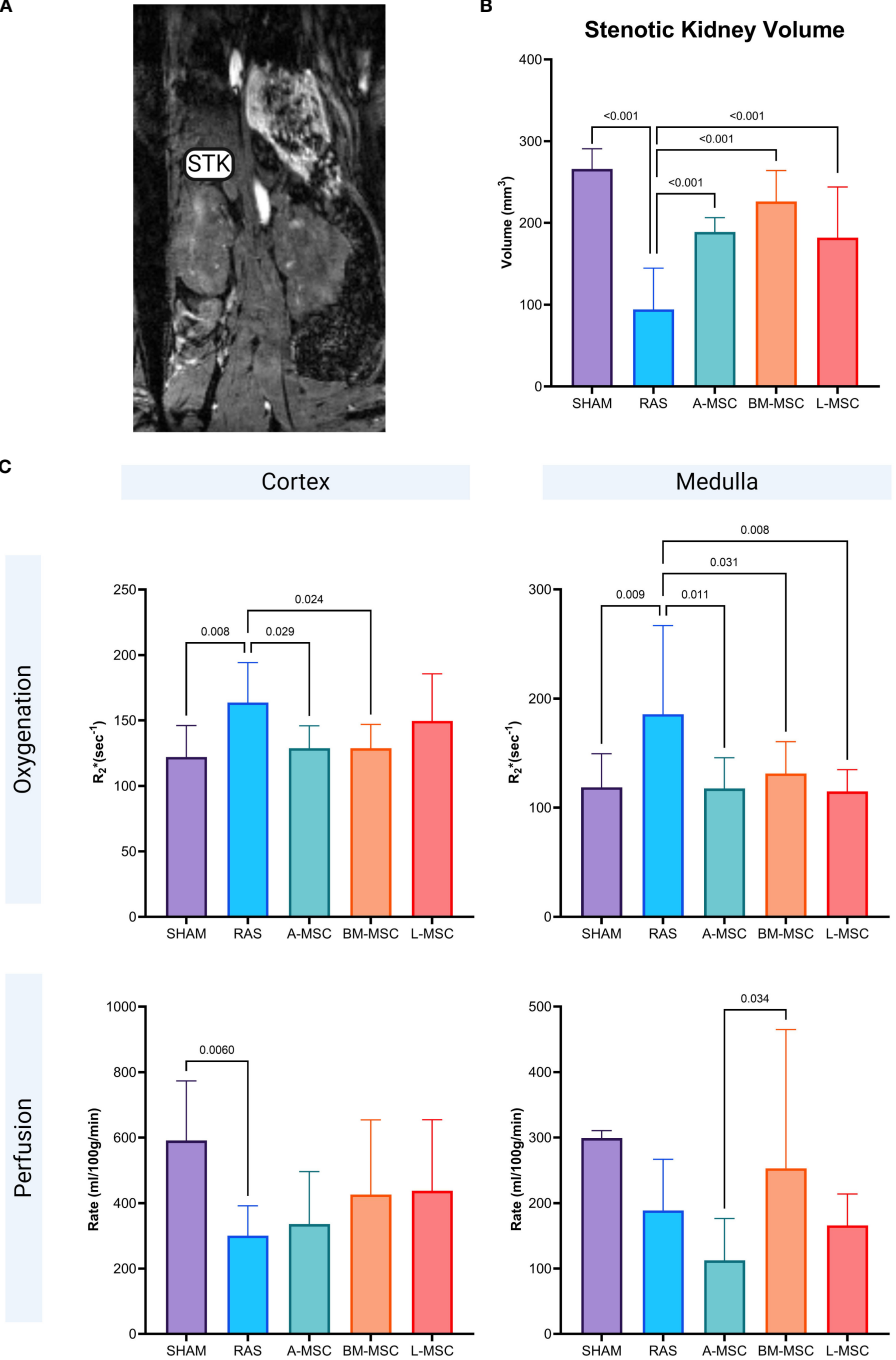
Representative MRI image of STK in coronal section **(A)**. Non-invasive measurement of volume in the STKs **(B)** and the oxygenation and perfusion to the cortex and medulla in the STKs **(C)** within each group. All measurements are expressed as mean ± SD. For oxygenation, R_2_*(sec-1) reflects hypoxia with lower R_2_* indicating better oxygenation. STK, stenotic kidney.

Non-invasive evaluation of the volume, perfusion, and oxygenation of the STKs were performed using micro-MRI analysis. Compared to the sham group, untreated RAS mice had significant loss of volume in the STKs (94.18 ± 50.6mm^3^ vs 266 ± 24.7mm^3^ in sham, p< 0.001), suggestive of ischemic injury (**Figures 2A, B**). With MSC treatment, the volumes of the STKs significantly improved compared to the untreated RAS mice (A-MSC: 188.8 ± 17.6mm^3^; BM-MSC: 226.1 ± 37.9mm^3^; L-MSC: 181.9 ± 62mm^3^; all vs RAS, p<0.001). No significant differences were noted in the volume of the STKs among the MSC treatment groups (**Figure 2B**). Cortical and medullary perfusion and oxygenation were also measured using micro-MRI. In this method, R_2_*(sec^-1^) reflects hypoxia, thus lower R_2_* indicated better oxygenation. The untreated RAS group had decreased oxygenation to both the cortex (163.8 ± 30.5 sec^-1^ vs 122.1 ± 24.1sec^-1^ in sham, p= 0.008) and the medulla (186 ± 81.3 sec^-1^ vs 119 ± 30.8 sec^-1^ in sham, p= 0.009) and decreased mean perfusion to the cortex (301 ± 91.2ml/100g/min vs 591 ± 182ml/100g/min in sham, p= 0.006) when compared to the sham group. Mice treated with MSC had higher oxygenation to the medullary region compared to the RAS group (A-MSC: 118 ± 28.2 sec^-1^, p= 0.011; BM-MSC: 131 ± 29.3 sec^-1^, p= 0.031; L-MSC: 115 ± 20 sec^-1^, p=0.008; all vs RAS), while those treated with A-MSC (128.8 ± 17.1 sec^-1^ vs 163.8 ± 30.5 sec^-1^ in RAS, p= 0.029) or BM-MSC (128.7 ± 18.2 sec^-1^ vs RAS, p= 0.024) had improved oxygenation in the cortex. No significant improvement was observed in perfusion to the cortex and medulla with MSC treatment (**Figure 2C**), but medullary perfusion in BM-MSC group was higher than in A-MSC group.

### Inflammatory profiles

**Figure 3:**
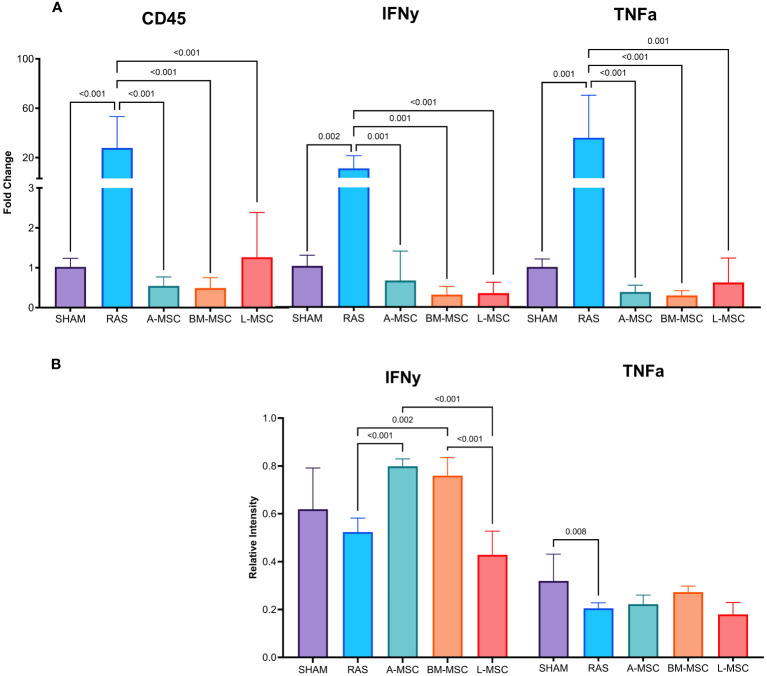
Levels of gene expression for overall inflammation (CD45), IFNy, and TNFa were measured using real-time PCR **(A)**. Protein expression of IFNy and TNFa were measured using western blot **(B)**. All measurements are expressed as mean ± SD. IFNy, interferon gamma; TNFa, tumor necrosis factor alpha.

Untreated RAS mice had significantly higher gene expression of CD45 (27.8 ± 25.5 vs 1.02 ± 0.2 in sham, p< 0.001), IFNy (10.8 ± 10.3 vs 1.03 ± 0.27 in sham, p= 0.002), and TNFa (35.9 ± 34.4 vs 1 ± 0.2 in sham, p= 0.001). Treatment with MSC of all types resulted in decreased gene expression of CD45 (A-MSC: 0.55 ± 0.23; BM-MSC: 0.49 ± 0.26; L-MSC: 1.26 ± 1.12; all vs RAS, p< 0.001); IFNy (A-MSC: 0.67 ± 0.74; BM-MSC: 0.31 ± 0.21; L-MSC: 0.35 ± 0.28; all vs RAS, p≤ 0.001); and TNFa (A-MSC: 0.39 ± 0.17; BM-MSC: 0.30 ± 0.12; L-MSC: 0.63 ± 0.61; all vs RAS, p≤ 0.001) when compared to the untreated RAS group (**Figure 3A**). On western blot, the protein expression of IFNy was higher for A-MSC (0.8 ± 0.03 vs RAS, p< 0.001) and BM-MSC treated mice (0.76 ± 0.07 vs RAS, p= 0.002) compared to untreated RAS mice (0.52 ± 0.06). On the other hand, mice treated with L-MSC (0.43 ± 0.1) had lower protein expression of IFNy compared to A-MSC (p< 0.001) and BM-MSC (p< 0.001) and similar level of expression to the untreated RAS group. No significant differences were observed for TNFa protein expression among untreated and MSC-treated RAS mice (**Figure 3B**), but they were no longer lower than sham.

**Figure 4:**
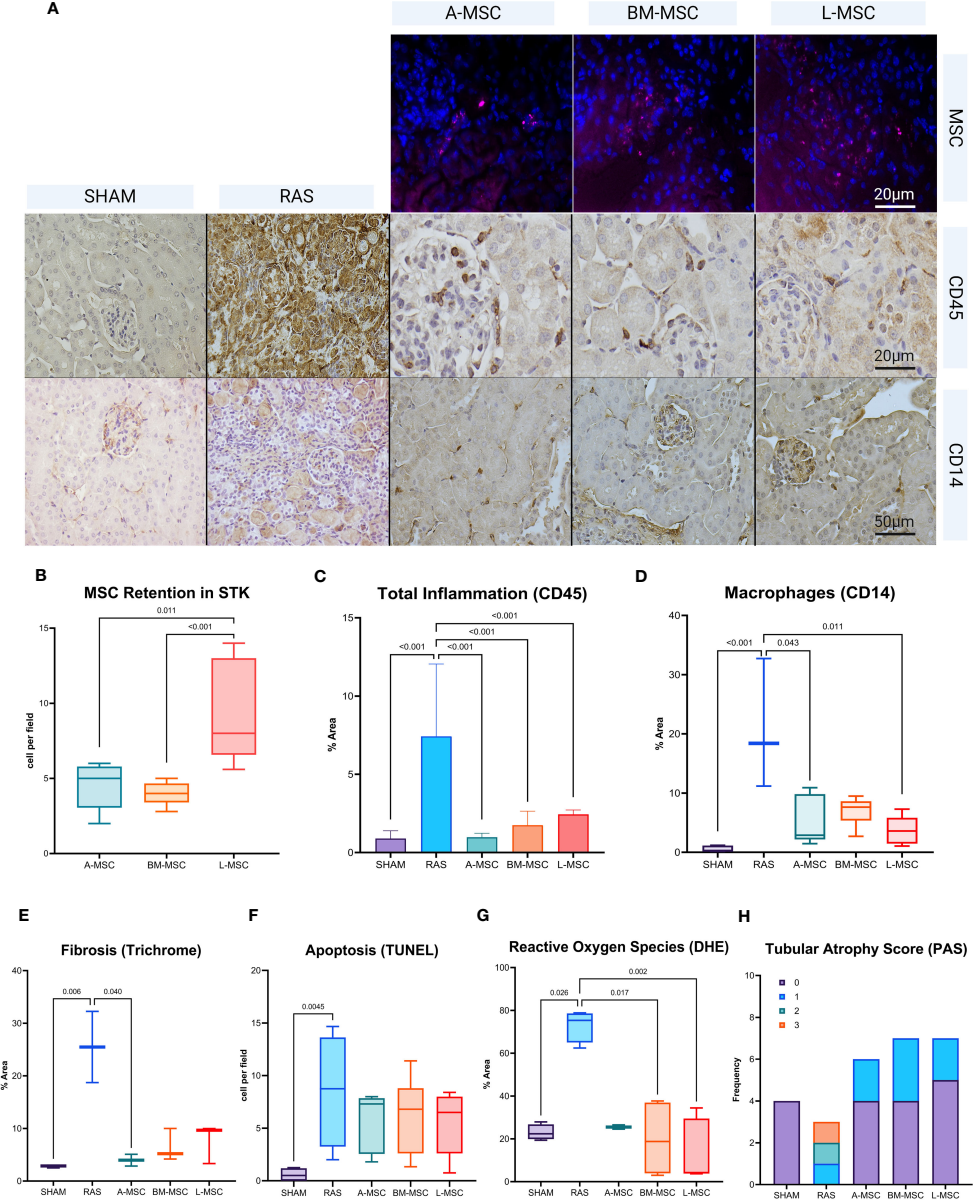
Representative histological images of MSC retention, CD45 stain, and CD14 stain. MSCs are labeled in pink. Positive staining for either CD45 or CD14 are in brown **(A)**. Manual counts (median ± IQR) of retained MSCs and TUNEL+ cells per high power field (40x) and percent area of positive stain for CD45 (mean ± SD), CD14 (median ± IQR), Trichrome (median ± IQR), DHE (median ± IQR), and PAS (counts in each score) in the STKs within each group are shown in **(B–H)**. MSC, mesenchymal stromal cells; TUNEL, Terminal deoxynucleotidyl transferase dUTP nick end labeling; DHE, Dihydroethidium; PAS, Periodic acid-Schiff.

MSC were tagged with a fluorescent protein (CTFR, in pink) prior to administration to allow for evaluation of their retention in the STK on unstained frozen sections. Among the three types, L-MSC (8 [6.4] cells) had the highest retention in the STK compared to A-MSC (5 [2.7] cells vs L-MSC, p= 0.011) or BM-MSC (4 [1.3] cells vs L-MSC, p< 0.001) (**Figures 4A, B**). Untreated RAS mice displayed the highest level of overall inflammation (CD45 positivity: 7.4 ± 4.6% vs 0.9 ± 0.5% in sham, p< 0.001) and total macrophage expression (CD14 positivity: 18.4 [21.5] % vs 0.2 [1] % in sham, p< 0.001) on histology compared to the sham group (**Figures 4A, C, D**). Infusion of all MSC types led to reduction in overall inflammation (A-MSC: 1 ± 0.3%; BM-MSC: 1.8 ± 0.9%; L-MSC: 2.4 ± 0.3%; all vs RAS, p< 0.001). For overall macrophage expression, A-MSC (2.9 [7.7] % vs RAS, p= 0.044) and L-MSC (3.6 [4.4] % vs RAS, p= 0.011) treated mice resulted in lower expression compared to untreated RAS mice (**Figures 4A, C, D**).

**Figure 5:**
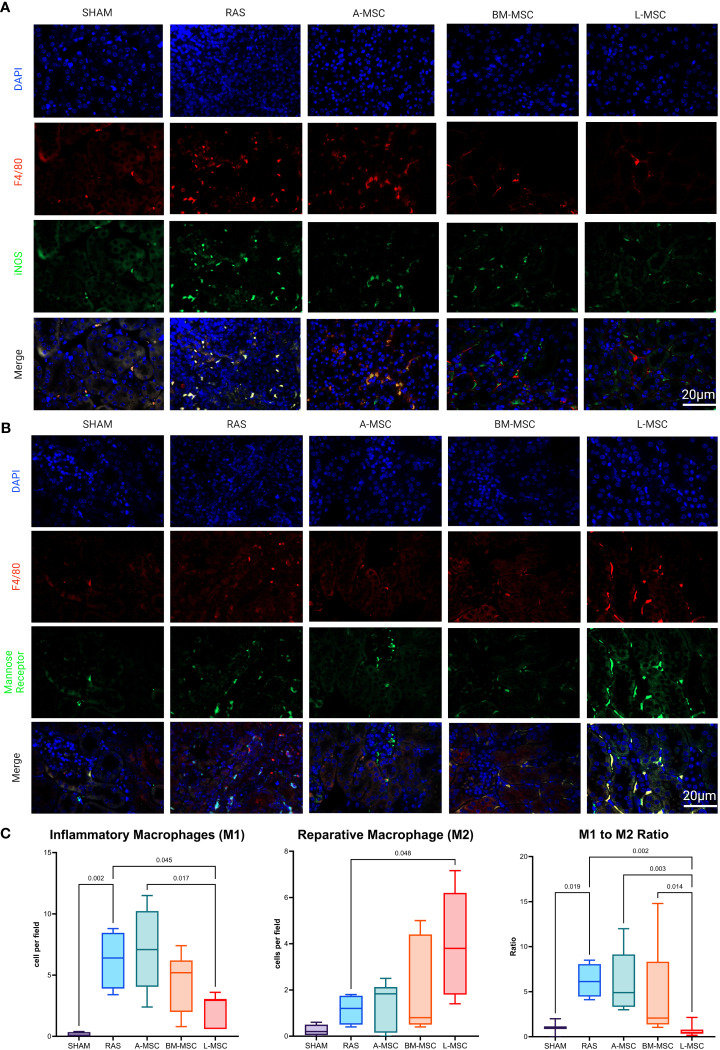
Representative images of inflammatory macrophages, M1 in **(A)** and reparative macrophages, M2 in **(B)** the STKs within each group. Manual counts of M1 and M2 per high power field (40x) and their ratio within the STKs are shown in **(C)** as median ± IQR. RAS, renal artery stenosis; A-MSC, Adipose mesenchymal stromal cells; BM-MSC, Bone Marrow mesenchymal stromal cells; L-MSC, Liver mesenchymal stromal cells.

Focusing specifically on M1 (inflammatory) and M2 (reparative) macrophage types, RAS led to significant increase in the frequency of M1 macrophages (6.4 [4.6] cells vs 0.1 [0.35] cells in sham, p= 0.002) in STKs (**Figures 5A, C**). L-MSC-treated mice had decreased frequency of M1 (3 [2.4] cells vs 6.4 [4.6] cells in RAS, p= 0.045) and markedly increased M2 macrophages (3.8 [4.4] cells vs 1.2 [1.3] cells in RAS, p= 0.048) in the STKs compared to untreated RAS mice (**Figures 5A–C**). Treatment with A-MSC or BM-MSC did not achieve significant reduction in M1 or elevation in M2 macrophages (**Figure 5C**). Looking at the ratio of M1 to M2 presence in the STKs, L-MSC-treated (ratio: 0.45 [0.46]) mice resulted in the lowest polarization toward the inflammatory M1 macrophage subtype compared to either untreated RAS (ratio: 6.13 [3.6] vs L-MSC, p= 0.002) or A-MSC (ratio: 4.9 [5.8] vs L-MSC, p= 0.003) and BM-MSC (ratio: 2.1 [7] vs L-MSC, p=0.014) treated mice (**Figure 5C**).

For non-immune related changes, significant reduction in fibrosis was noted for A-MSC (4 [2.3]% vs 25.5 [13.5]% RAS, p= 0.04) treated mice. Oxidative stress was also reduced for BM-MSC (18.8 [33.1]% vs RAS, p= 0.017) and L-MSC (4.3 [25.7]% vs RAS, p= 0.002) treated mice compared to untreated RAS group (75.3 [13.6]%). No significant differences were noted in apoptosis or tubular atrophy scores between untreated RAS and MSC treated groups (**Figures 4E–H**).

## Discussion

In this study, we aimed to characterize the effect of the novel L-MSC *in-vivo* and to directly compare their impact on ischemic injury to the more established A- and BM-MSC. We demonstrated that L-MSC are equally as effective as A-MSC and BM-MSC at improving renal function, the volume, and oxygenation of the renal medulla in the STKs. Additionally, L-MSC-treated RAS mice achieved a similar reduction in inflammation in the STKs as those treated with A- and BM-MSC. However, significantly more L-MSC were retained in the STKs, and L-MSC-treated mice had greater polarization of macrophages toward a more reparative (M2) phenotype compared to A- and BM-MSC treated groups.

MSC have been extensively investigated as therapeutic agents for inflammatory conditions, classically in graft versus host disease and inflammatory bowel disease, but also in ischemic renal injury (9–12). Although the clinical efficacy of MSC treatments has been variable, data from experimental and human clinical trials support MSCs’ immunomodulatory potential through intricate communications with both the innate and adaptive immune system via several proposed routes, including paracrine secretions, direct cell-to-cell contact, and release of exosomes. The downstream effect is the resolution of inflammation and the promotion of tissue regeneration through several mediatory pathways such as induction of M2 macrophage polarization (12, 13).

The source tissue of MSC and the microenvironment in which they are found impact MSC functions and properties. The liver is often considered to be an immunologically privileged organ that serves as a critical immune interface (14). Several clinical studies involving simultaneous liver and kidney transplant or simultaneous liver and heart transplant demonstrate that compared to solitary kidney or heart transplants, the presence of concomitant liver allograft was protective against both T cell and antibody-mediated rejection and overall improved graft survival (15–18). On a cellular level, simultaneous liver and kidney transplant recipients demonstrated lower frequency of circulating CD8^+^, activated CD4^+^, and effector memory T cells and had decreased alloreactivity to donor cells compared to solitary kidney transplant recipients (16). Likewise, secretome analysis of simultaneous liver and kidney transplant recipients showed downregulation of inflammatory pathways and upregulation of tissue integrity pathways (17). Taken together, the superior immunomodulatory properties of the MSC isolated from liver may be closely associated with the immune context surrounding the organ.

Our findings in this study underscore previous studies that demonstrated improvement in renal function, oxidative stress, and inflammation after MSC treatment (4, 19, 20) as well as the impact of MSC on macrophage polarization (12). However, the current study augments the previous bodies of literature in several ways. We directly determined and compared the positive impact of MSC isolated from liver tissue, which has not been explored in detail to our best knowledge as a therapeutic agent, to that of more established MSC isolated from adipose and bone marrow tissues. Additionally, we demonstrated that a significantly higher number of L-MSC homed to site of injury than A-MSC and BM-MSC and exhibited greater impact on macrophage phenotypes. Interestingly, for more structural related changes, only A-MSC treated group achieved reduction in fibrosis while BM- and L-MSC treated groups showed significant reduction in levels of reactive oxygen species. While MSCs generally share many similar characteristics, previous studies have demonstrated significant differences among A-, BM, and L-MSCs that may explain some of the differing effects we observed in this study. For example, *in-vitro* studies have shown that L-MSC have a more homogenous migration kinetics toward chemoattractants than A-MSC, while the latter have superior anti-fibrotic and pro-angiogenic properties (21–24). Macrophage polarization plays a major role in liver disease (25). M1 macrophages promote tissue injury in vast majority of the liver diseases (viral, alcohol-related and metabolic-associated), whereas M2 macrophages attenuate liver injury and inflammation (26). At steady state, the liver microenvironment favors M2 polarization (27) for homeostasis. Interestingly, here, we demonstrate that adoptive transfer of human L-MSC in a mouse model of inflammation also promotes M2 polarization. Thus, it is possible that L-MSC have a role in liver homeostasis, which will need to be investigated further in the future.

Our study is not without limitations. The MSC treated groups did not result in improvement in blood pressures and perfusion or decrease in apoptosis compared to untreated RAS group. Given our small sample size, it is possible that our study may not have been adequately powered to evaluate all these physiological and histological changes. We also found that despite L-MSC-treated RAS mice having lower plasma renin, the urinary protein level was higher compared to the A-MSC group. This might be due to differential impact of MSC types on cells in the juxtaglomerular apparatus. Additionally, we noted discordance between IFNy gene expression and protein expression for A- and BM-MSC treated mice. The elevated IFNy protein expression in the A- and BM-MSC groups, but not in L-MSC group, could be related to post-transcriptional regulation. Indeed, previous transcriptomic analysis comparing A-, BM-, and L-MSC demonstrated significant upregulation of INFy regulatory genes in L-MSC (1, 2), further supporting that L-MSC likely exert greater influence on the immune system than A- or BM-MSC. Additionally, while some of the superior effects on macrophages might have resulted from the engraftment of a larger number of L-MSC compared to A- and BM-MSC, such differences were not consistently observed in other parameters. Therefore, cell number may not have been the sole determinant of L-MSCs’ effects. In our study, mice were also given a single infusion of MSC. Multiple infusions may be needed in order for MSC to exert maximal effect on the ischemic injury to the kidney (28). Additionally, more time than the allocated two weeks in this study may have been needed to see a more pronounced impact of reduced inflammation on renal function in the MSC-treated groups.

In summary, our study established the effect of L-MSC *in-vivo* on ischemic injury and directly compared their impact to that of A-MSC and BM-MSC. We showed that L-MSC are as effective as the commonly studied A- and BM-MSC at mitigating ischemic renal injuries. Furthermore, they are superior at homing to site of injury and at inducing polarization toward reparative macrophages when compared to A- and BM-MSC. Based on these findings, we are currently exploring the effect of local delivery of MSC on alloimmune mediated damages through direct infusion into the allograft renal artery in our ongoing clinical trials with adult renal transplant recipients (NCT05456243). As part of the clinical trial, we are collaborating with the Mayo Clinic Center for Regenerative Biotherapeutics Laboratory (IRB 17-007379) to routinely generate and culture MSC cell lines from adipose, bone marrow, and liver tissue (1cm x 1cm biopsy sample) from healthy adult donors in a GMP facility and testing for MSC phenotypic markers and tri-lineage differentiation to meet the release criteria for clinical use. More work will need to be done to detail the mechanism(s) through which L-MSC interact with the immune system to effectuate their impact on the surrounding environment.

## Data availability statement

The raw data supporting the conclusions of this article will be made available by the authors, without undue reservation.

## Ethics statement

The studies involving humans were approved by Mayo Clinic Institutional Review Board. The studies were conducted in accordance with the local legislation and institutional requirements. The human samples used in this study were acquired from primarily isolated as part of your previous study for which ethical approval was obtained. Written informed consent for participation was not required from the participants or the participants’ legal guardians/next of kin in accordance with the national legislation and institutional requirements. The animal study was approved by Institutional Animal Care and Use. The study was conducted in accordance with the local legislation and institutional requirements.

## Author contributions

YL: Conceptualization, Data curation, Formal analysis, Investigation, Methodology, Writing – original draft, Writing – review & editing. EO: Conceptualization, Data curation, Formal analysis, Investigation, Methodology, Writing – review & editing. MH: Data curation, Formal analysis, Investigation, Writing – review & editing. HT: Data curation, Formal analysis, Investigation, Writing – review & editing. IS: Data curation, Formal analysis, Investigation, Writing – review & editing. KJ: Data curation, Formal analysis, Investigation, Writing – review & editing. JK: Data curation, Formal analysis, Investigation, Writing – review & editing. DG: Data curation, Formal analysis, Investigation, Writing – review & editing. JG: Data curation, Formal analysis, Investigation, Writing – review & editing. LL: Conceptualization, Formal analysis, Investigation, Methodology, Writing – review & editing. TT: Conceptualization, Data curation, Formal analysis, Investigation, Methodology, Writing – review & editing.
